# Information-Entropic Deep Learning with Gaussian Process Regularisation for Uncertainty-Aware Quantitative Trading

**DOI:** 10.3390/e28050485

**Published:** 2026-04-23

**Authors:** Feng Lin, Huaping Sun

**Affiliations:** 1 School of Materials Science and Engineering, University of Science and Technology Beijing, Beijing 100083, China; u202340335@xs.ustb.edu.cn; 2 School of Economics and Management, University of Science and Technology Beijing, Beijing 100083, China

**Keywords:** information entropy, quantitative trading, Gaussian process, deep learning, uncertainty quantification, risk management, portfolio optimisation, semiparametric regression, CVaR, Kelly criterion, predictive entropy, Kullback–Leibler divergence, differential entropy

## Abstract

Quantitative trading systems require predictive models that simultaneously deliver accurate forecasts, calibrated uncertainty quantification, and actionable risk measures. This paper proposes an information-theoretic semiparametric regression framework combining a convolutional neural network–Transformer (CNN–Transformer) network for nonlinear temporal dependencies with a Gaussian process (GP) prior for residual autocorrelation and calibrated predictive distributions. Three theoretical results are established: an identifiability theorem guarantees joint recoverability of the nonparametric and GP components; a consistency theorem showing that the penalised maximum likelihood estimator converges at a rate $n^{-1/(2+d_{\mathrm{eff}})}$; and a coverage theorem proving asymptotic nominal coverage of the GP’s credible intervals. The framework enables an entropy-regulated trading module where predictive differential entropy informs position sizing via an uncertainty-penalised Kelly criterion, Kullback–Leibler divergence quantifies model uncertainty, and CVaR-constrained optimisation controls the tail risk. Simulations show the method outperforms the CNN, long short-term memory (LSTM), Transformer, XGBoost, random forest, least absolute shrinkage and selection operator (LASSO), and standard GP regression approaches. Backtesting on four Chinese A-share stocks yielded annualised returns of 15.9–22.4% with Sharpe ratios of 0.49–0.62, maximum drawdowns below 15%, and daily 95% CVaR reductions of 28–31% relative to a full-Kelly baseline, confirming both predictive accuracy and risk management effectiveness.

## 1. Introduction

Stock price prediction must capture nonlinear, non-stationary temporal dynamics, provide calibrated uncertainty measures, and translate them into risk management decisions. Existing approaches satisfy at most two simultaneously.

From an information-theoretic view, the differential entropy of a predictive distribution quantifies irreducible forecast uncertainty; low entropy warrants larger positions, and high entropy demands caution. Shannon [1] established entropy as the unique uncertainty measure, and Kelly [2] showed that the optimal growth rate strategy equals the mutual information between the predictor and return. Despite this, modern trading systems rarely exploit information-theoretic quantities. We bridge this gap using the GP predictive differential entropy and KL divergence between kernel models for uncertainty quantification and risk-aware position sizing. This connects to broader efforts showing that Bayesian inference and stochastic optimisation can improve real-world trading [3].

For point prediction, ARMA [4] and ARIMA [5] suit stationary series but fail under non-stationarity [3]. Decision trees [6], random forests [7], XGBoost [8], and LightGBM [9] models capture nonlinear relationships but require manual temporal features. LSTM models [10,11] model long-range dependencies, CNNs [12,13,14] extract local patterns, and Transformers [15,16] capture global interactions; hybrids of these [12,13] are promising. All produce point estimates without principled uncertainty quantification.

For uncertainty quantification, Bayesian neural networks provide posteriors but are costly and hard to calibrate [17]; MC dropout [18] lacks coverage guarantees; and GP regression [19] gives exact calibrated intervals but scales poorly and cannot model complex financial feature interactions [20]. Crucially, the GP posterior yields a closed-form differential entropy, unlike BNNs and MC dropout, which require sampling-based entropy estimates.

For risk-aware decisions, the Kelly criterion [2] and CVaR-constrained optimisation [21] both require the full predictive distribution, yet existing studies use simple threshold rules on point forecasts [22,23,24,25]. The Kelly optimal growth rate equals the mutual information between the prediction and return [2,26], but our framework operationalises this by modulating position sizes via GP predictive entropy.

Table 1 contrasts the proposed framework with representative existing studies across six key dimensions, including theoretical guarantees.

As Table 1 shows, no existing method simultaneously addresses all six dimensions. The proposed framework fills this gap by integrating deep feature extraction, Gaussian process uncertainty quantification, information-entropic risk measures, risk-aware trading, and theoretical consistency guarantees within a single semiparametric regression model that generalises the classical partially linear framework of Robinson [28].

The main contributions form a progressive chain. We emphasise that the individual tools (CNNs, Transformers, and Gaussian processes) are well established. The novelty lies in their integration within a semiparametric framework with explicit information-theoretic structural consequences and in the application to risk-aware quantitative trading. The semiparametric deep Gaussian process regression model (CNN–Transformer–Gaussian process regression (C-T-GPR)) decomposes stock returns into a deterministic component modelled by a CNN-Transformer network and a stochastic component modelled by a Gaussian process prior, with theoretical guarantees for consistency, convergence rates, and asymptotic nominal coverage of GP credible intervals. An entropy-regulated trading module decomposes predictive differential entropy into aleatoric and epistemic components, derives an entropy-penalised Kelly criterion, and formulates CVaR-constrained portfolio optimisation controlling tail risk via the full predictive distribution.

The contribution is integrative rather than component-level; combining deep learning, Gaussian processes, information theory, and portfolio optimisation within a semiparametric framework yields structural consequences (entropy decomposition, entropy-penalised Kelly sizing, and epistemic uncertainty indexing) which are unavailable when components are used in isolation. Theoretical results guarantee asymptotic consistency of entropy-based risk quantities, justifying their use in trading decisions. Experiments test this integrative claim, supplemented by adversarial simulations, sub-period robustness analysis, finite-sample calibration diagnostics, baseline tuning protocols, and transaction cost sensitivity analysis.

The remainder of this paper is organised as follows. Section 2 develops the semiparametric framework, including the model formulation, the CNN-Transformer architecture, the Gaussian process prior, the penalised MLE, and the theoretical results. Section 3 develops the information-theoretic analysis, including predictive entropy decomposition and its connection to financial risk. Section 4 derives the entropy-regulated trading strategy. Section 5 reports the simulation and real-data experiments. Section 6 discusses the implications and limitations. Section 7 concludes the paper.

## 2. Semiparametric Deep Gaussian Process Framework

### 2.1. Model Formulation

Consider a matrix-valued time series predictor $X\in R^{T\times D_{1}}$, whose columns $x_{d}={\left(x_{d,1},\ldots ,x_{d,T}\right)}^{⊤}\in R^{T}$, $d=1,\ldots ,D_{1}$, are *T*-period univariate series (e.g., closing prices, trading volumes, and macroeconomic indicators) such that the entry $x_{d,t}$ denotes the value of feature *d* at lag *t*. In addition, let $Z\in R^{D_{2}}$ be a vector-valued predictor encoding factors associated with the temporal autocorrelation of the response (e.g., lagged returns, momentum indicators, and volatility measures), with components $z_{j}\in R$ for $j=1,\ldots ,D_{2}$. Throughout the paper, bold lowercase letters denote (column) vectors, bold uppercase letters denote matrices, and unbolded italic letters denote scalars.

**Definition** **1** (Semiparametric stock return model)**.** *The stock return Y satisfies*

(1) Y=f(X)+g(Z)+ε,Y∈R,

*Here, Y is a scalar response (the next period’s return), $X\in R^{T\times D_{1}}$ and $Z\in R^{D_{2}}$ are the predictors defined above, and bold notation is used consistently for vector- and matrix-valued quantities, where $f:R^{T\times D_{1}}\to R$ is a deterministic function capturing nonlinear feature interactions, $g∼\mathrm{GP}(0,k_{\theta })$ is a Gaussian process modelling residual autocorrelation and temporal random effects, $\varepsilon ∼N(0,\sigma^{2})$ is independent observation noise, and (f,g,ε) are mutually independent.*

**Remark** **1** (Information-theoretic interpretation of the decomposition)**.** *Throughout, H(·) denotes the differential (Shannon) entropy, $D_{\mathrm{KL}}(\cdot \;∥\;\cdot )$ denotes the Kullback–Leibler divergence, and I(·;·) denotes the mutual information, all measured in nats (natural logarithm units).*

*The decomposition in Equation *(1)* admits a natural information-theoretic reading. The deterministic component f captures the mutual information I(Y;X)=H(Y)−H(Y∣X) between the response and the observable features, while the GP component g captures the residual mutual information I(Y−f(X);Z) between the residual and the autocorrelation-inducing predictors. The noise ε represents the irreducible aleatoric entropy $H\left(\varepsilon \right)=\frac{1}{2}\ln\left(2\pi e\sigma^{2}\right)$. Maximising the marginal likelihood of the model in Equation *(1)* is equivalent to minimising the KL divergence between the empirical distribution and the model, ensuring that the information content of the data is optimally allocated between the parametric (f) and nonparametric (g) components.*

**Assumption** **1.** (A1) *Here, f belongs to the Sobolev ball $W^{s,2}\left(B\right):=\{h\in L^{2}\left(R^{D_{1}}\right){:∥h∥}_{W^{s,2}}\leq B\}$, where the Sobolev norm is defined via weak partial derivatives as ${∥h∥}_{W^{s,2}}^{2}=\sum_{|\alpha |\leq s}\int_{R^{D_{1}}}{\left|D^{\alpha }h\left(x\right)\right|}^{2}\;dx$, the smoothness index satisfies $s>D_{1}/2$ (ensuring $W^{s,2}↪C^{0}$ via the Sobolev embedding theorem), and B>0 is a fixed radius. This is the standard $L^{2}$-based Sobolev space, and the convergence rate in Theorem 2 depends on s through the embedding constant.*

(A2) *The kernel $k_{\theta }$ is stationary, continuous, and satisfies $k_{\theta }\left(0\right)=\sigma_{g}^{2}<\infty$ for all θ∈Θ, where *Θ* is compact.*
(A3) *The predictors $\left(X_{i},Z_{i}\right)$ are strictly stationary and α-mixing, with the mixing coefficients satisfying $\sum_{k=1}^{\infty }\alpha {\left(k\right)}^{\delta /(2+\delta )}<\infty$ for some δ>0.*
(A4) *$E[|Y{|}^{2+\delta }]<\infty$, and the marginal density of Z is bounded away from zero on its support.*

**Theorem** **1** (Identifiability)**.** *Under Assumption 1, the decomposition Y=f(X)+g(Z)+ε is identifiable in the sense that if $f_{1}\left(X\right)+g_{1}\left(Z\right)=f_{2}\left(X\right)+g_{2}\left(Z\right)$ almost surely, then $f_{1}=f_{2}$ almost surely, and $g_{1}=g_{2}$ in distribution.*

**Proof.**  Taking the conditional expectation yields E[Y∣X]=f(X)+E[g(Z)∣X]. By Assumption 1 (A5), X and Z are independent, and since *g* is a zero-mean Gaussian process driven by Z and independent of both X and Z, we have E[g(Z)∣X]=E[g(Z)]=0. Hence f(X)=E[Y∣X] is uniquely determined. Given *f*, the residual g(Z)=Y−f(X)−ε has a law determined by the covariance structure of Y−f(X) conditional on Z, since ε is white noise independent of Z. This determines *g* in distribution.    □

### 2.2. CNN-Transformer Feature Extraction: $f_{\eta }$

The deterministic component *f* is approximated by a CNN-Transformer network $f_{\eta }:R^{T\times D_{1}}\to R$, where $\eta \in R^{p_{n}}$ collects all trainable weights and biases of the network, namely the convolutional kernels ${\left\{W^{\left(k\right)},b^{\left(k\right)}\right\}}_{k=1}^{N}$ in Equation (2), the squeeze-and-excitation gating matrices $W_{1},W_{2}$ in Equation (4), the multi-head attention projection matrices ${\left\{W_{Q_{h}},W_{K_{h}},W_{V_{h}}\right\}}_{h=1}^{H}$ and output projection $W_{O}$ in Equations (5)–(7), and the parameters of the position-wise feedforward and final fully connected layers. The total parameter count $p_{n}$ is allowed to grow with the sample size *n*, as formalised in Assumption 2(B1). Hyperparameters that are not contained in η (number of layers, kernel sizes, number of attention heads, and hidden dimension) are treated as fixed structural choices. The architecture consists of three modules: domain-specific CNN feature extraction, Transformer cross-feature fusion, and a fully connected prediction head.

CNN module. Task-specific 1D convolutional sub-networks extract local temporal patterns from each data type (macroeconomic, market transaction, and fundamental data). For input $X\in R^{T}$, the *k*th convolutional layer produces

(2) $$H^{\left(k\right)}=\sigma \left(W^{\left(k\right)}*H^{\left(k-1\right)}+b^{\left(k\right)}\right),\;k=1,\ldots ,N,$$

where $H^{\left(0\right)}=X$ is the input (and for k≥1, the input to layer *k* is the pooled output $P^{\left(k-1\right)}$ from the previous layer), $H^{\left(k\right)}$ is the feature map after the *k*th convolutional layer, $W^{\left(k\right)}$ is the learnable kernel tensor of layer *k* with a shape $\left(c_{k-1},c_{k},\ell_{k}\right)$ ($c_{k-1}$ and $c_{k}$ are the input and output channel counts, respectively, and $\ell_{k}$ is the temporal kernel length), $b^{\left(k\right)}\in R^{c_{k}}$ is the bias, σ(·)=max(0,·) is the ReLU activation, ∗ denotes 1D temporal convolution along the time axis, and *N* is the number of convolutional layers. A max-pooling operation with a stride *s* and window size *U* follows each layer:

(3) $$P^{\left(k\right)}\left[i,:\right]=\max\left\{H^{\left(k\right)}\left[s\cdot i+m,:\right]|m=0,\ldots ,U-1\right\}.$$

Here, $P^{\left(k\right)}\left[i,:\right]$ denotes the *i*th row (timestep *i*) of the post-pooling feature map, and the maximum is taken over the *U* consecutive temporal positions {s·i,s·i+1,…,s·i+U−1}. After *N* convolutional blocks, the aggregated CNN output across all sub-networks is $H_{\mathrm{cnn}}\in R^{T^{\prime }\times d}$, where $T^{\prime }=\left\lfloor T/s^{N}\right\rfloor$ is the reduced temporal length and *d* is the total channel dimension.

Channel attention. A squeeze-and-excitation mechanism [29] reweights channels to suppress noise and enhance discriminative features. Global average pooling along the temporal axis yields channel descriptors $M_{c}=T^{\prime -1}\sum_{t=1}^{T^{\prime }}H_{c}\left(t\right)$, where $T^{\prime }$ is the reduced temporal length after convolution and pooling and *C* is the number of channels (i.e., the output dimension *d* of the CNN module). A gating network produces attention weights

(4) $$S=\sigma_{\mathrm{sig}}\left(W_{2}\cdot \mathrm{ReLU}\left(W_{1}M\right)\right)\in R^{C},$$

where $M={\left(M_{1},\ldots ,M_{C}\right)}^{⊤}\in R^{C}$, $W_{1}\in R^{(C/r)\times C}$, and $W_{2}\in R^{C\times (C/r)}$ are learnable weight matrices with a reduction ratio *r* and $\sigma_{\mathrm{sig}}$ is the sigmoid function. The refined features are ${\tilde{H}}_{c}\left(t\right)=S_{c}\cdot H_{c}\left(t\right)$ for each channel *c* and timestep *t*.

**Remark** **2** (Entropy interpretation of channel attention)**.** *The squeeze-and-excitation mechanism can be viewed as an information-theoretic channel selector. The sigmoid gate $S_{c}$ approximates the posterior probability that channel c carries relevant information, and the reweighting ${\tilde{H}}_{c}=S_{c}\cdot U_{c}$ implements a soft information bottleneck [30] that suppresses high-entropy (noisy) channels and preserves low-entropy (informative) ones, maximising the mutual information $I(\tilde{H};Y)$ between the refined features and the target.*

Transformer module. Positional encoding is added to the refined CNN output: $E={\tilde{H}}_{\mathrm{cnn}}+\mathrm{PosEnc}$. Multi-head self-attention (MHA) captures cross-feature correlations:

(5) $$\begin{array}{cc} Q_{h} & =EW_{Q_{h}},\;K_{h}=EW_{K_{h}},\;V_{h}=EW_{V_{h}}, \end{array}$$

(6) $$\begin{array}{cc} {\mathrm{head}}_{h} & =\mathrm{softmax}\;\left(\frac{Q_{h}K_{h}^{⊤}}{\sqrt{d_{k}}}\right)V_{h}, \end{array}$$

(7) $$\begin{array}{cc} \mathrm{MHA} & =\mathrm{Concat}\left({\mathrm{head}}_{1},\ldots ,{\mathrm{head}}_{H}\right)\;W_{O}. \end{array}$$

**Remark** **3** (Attention entropy as an information flow indicator)**.** *The softmax attention weights $\alpha_{ij}=\mathrm{softmax}{\left(Q_{h}K_{h}^{⊤}/\sqrt{d_{k}}\right)}_{ij}$ define a probability distribution over the key positions. The attention entropy $H_{\mathrm{attn}}=-\sum_{j}\alpha_{ij}\ln\alpha_{ij}$ quantifies how broadly or narrowly the model distributes attention. Low attention entropy indicates focused information extraction from a few key timesteps, while high attention entropy suggests diffuse, uncertain relevance, a signal that can be propagated to the trading module as an additional epistemic uncertainty indicator.*

A position-wise feedforward network (FFN) with residual connections and layer normalisation yields the Transformer output $H_{\mathrm{trans}}\in R^{T^{\prime }\times d}$. Max pooling, vectorisation, and a fully connected layer produce the scalar prediction

(8) $$f_{\eta }\left(X\right)=\sigma_{o}\circ \mathrm{FC}\circ \mathrm{Transformer}\circ \mathrm{CNN}\left(X\right).$$

**Remark** **4** (Universal approximation)**.** Under standard conditions for the width and depth [31,32], the CNN-Transformer network $f_{\eta }$ is a universal approximator. For any ϵ>0 and $f\in W^{s,2}\left(B\right)$, there exists $\eta^{*}$ such that $∥f_{\eta^{*}}{-f∥}_{L^{2}}<\epsilon$. This ensures that the parametric approximation error can be made arbitrarily small.

### 2.3. Gaussian Process Prior with Dynamic Kernel Learning

The stochastic component *g* is endowed with a Gaussian process prior:

(9) $$g\left(Z\right)∼\mathrm{GP}\left(0,\;k_{\theta }\left(Z,Z^{\prime }\right)\right).$$

The choice of kernel $k_{\theta }$ determines the smoothness and correlation structure of *g*. We consider a mixture kernel that adapts to heterogeneous market regimes:

(10) $$k_{\theta }\left(Z,Z^{\prime }\right)=w_{1}\;k_{\mathrm{SE}}\left(Z,Z^{\prime };\ell_{1}\right)+w_{2}\;k_{\mathrm{Mat}}\left(Z,Z^{\prime };\nu ,\ell_{2}\right)+w_{3}\;k_{\mathrm{Per}}\left(Z,Z^{\prime };p,\ell_{3}\right),$$

where $k_{\mathrm{SE}}$ is the squared exponential kernel capturing smooth variations, $k_{\mathrm{Mat}}$ is the Matérn kernel capturing rough local fluctuations, $k_{\mathrm{Per}}$ is a periodic kernel capturing cyclical patterns (e.g., monthly effects), and $\theta =(w_{1},w_{2},w_{3},\ell_{1},\ell_{2},\ell_{3},\nu ,p)$ are jointly estimated via marginal likelihood. The individual kernels are

(11) $$\begin{array}{cc} k_{\mathrm{SE}}\left(Z,Z^{\prime };\ell \right) & =\sigma_{1}^{2}\exp\;\left(-\frac{∥Z-Z^{\prime }{∥}^{2}}{2\ell^{2}}\right), \end{array}$$

(12) $$\begin{array}{cc} k_{\mathrm{Mat}}\left(Z,Z^{\prime };\nu ,\ell \right) & =\sigma_{2}^{2}\frac{2^{1-\nu }}{\Gamma (\nu )}{\left(\frac{\sqrt{2\nu }∥Z-Z^{\prime }∥}{\ell }\right)}^{\nu }B_{\nu }\;\left(\frac{\sqrt{2\nu }∥Z-Z^{\prime }∥}{\ell }\right), \end{array}$$

(13) $$\begin{array}{cc} k_{\mathrm{Per}}\left(Z,Z^{\prime };p,\ell \right) & =\sigma_{3}^{2}\exp\;\left(-\frac{2\sin^{2}(\pi ∥Z-Z^{\prime }∥/p)}{\ell^{2}}\right). \end{array}$$

The mixture weights $w_{i}>0$ with $\sum_{i}w_{i}=1$ are optimised alongside all other hyperparameters, enabling automatic selection of the dominant kernel component based on the data. This is a key difference from standard GPR, where a single kernel family is pre-specified.

Let $M=\{k^{\left(1\right)},k^{\left(2\right)},k^{\left(3\right)}\}$ denote the candidate kernel components (SE, Matérn, and periodic). The posterior model weight for component *m* is

(14) $$\pi \left(m|D\right)=\frac{p\left(D|M_{m}\right)\;\pi \left(m\right)}{\sum_{m^{\prime }}p\left(D|M_{m^{\prime }}\right)\;\pi \left(m^{\prime }\right)},$$

where π(m) is the prior probability assigned to kernel component *m* before observing the data. We adopt the uninformative uniform prior π(m)=1/3 for m∈{SE,Mat,Per}, reflecting the absence of a priori preference among the squared-exponential, Matérn, and periodic kernels, and $p(D|M_{m})$ is the marginal likelihood under kernel *m*, computed in closed form for Gaussian processes as follows:

(15) $$\log p\left(D|M_{m}\right)=-\frac{1}{2}r^{⊤}C_{m}^{-1}r-\frac{1}{2}\log\left|C_{m}\right|-\frac{n}{2}\log\left(2\pi \right),$$

with $r=Y-f_{\hat{\eta }}$ and $C_{m}=K_{m}+{\hat{\sigma }}^{2}I$.

**Remark** **5** (KL divergence interpretation of model selection)**.** *The marginal likelihood *(15)* is directly related to the negative KL divergence between the data-generating distribution and the model. Model m receives a higher posterior weight π(m∣D) when its KL divergence from the true residual process is smaller. The BMA predictive distribution *(16)* therefore minimises the expected KL divergence from the true predictive distribution over the model space M, providing an information-theoretically optimal combination of kernel components.*

The BMA predictive distribution is then

(16) $$p\left(Y^{*}|D\right)=\sum_{m=1}^{3}\pi \left(m|D\right)\;p\left(Y^{*}|D,M_{m}\right),$$

which is a Gaussian mixture with a mean and variance:

(17) $$\begin{array}{cc} {\hat{\mu }}_{\mathrm{BMA}}^{*} & =\sum_{m}\pi_{m}\;{\hat{\mu }}_{m}^{*}, \end{array}$$

(18) $$\begin{array}{cc} {\hat{v}}_{\mathrm{BMA}}^{*} & =\sum_{m}\pi_{m}\left({\hat{v}}_{m}^{*}+{\left({\hat{\mu }}_{m}^{*}\right)}^{2}\right)-{\left({\hat{\mu }}_{\mathrm{BMA}}^{*}\right)}^{2}. \end{array}$$

The BMA variance (18) decomposes into the within-model uncertainty ($\sum_{m}\pi_{m}{\hat{v}}_{m}^{*}$) and between-model uncertainty ($\sum_{m}\pi_{m}{\left({\hat{\mu }}_{m}^{*}-{\hat{\mu }}_{\mathrm{BMA}}^{*}\right)}^{2}$), capturing an additional source of epistemic uncertainty that is invisible to single-kernel methods. This between-model term directly enters the entropy-penalised Kelly criterion (36), making position sizing more conservative when the data does not clearly favour one kernel over another.

### 2.4. Penalised Maximum Likelihood Estimation

Given the training data ${\left\{X_{i},Z_{i},Y_{i}\right\}}_{i=1}^{n}$, we define $Y={\left(Y_{1},\ldots ,Y_{n}\right)}^{⊤}$, and $f_{\eta }={\left(f_{\eta }\left(X_{1}\right),\ldots ,f_{\eta }\left(X_{n}\right)\right)}^{⊤}$, while $K_{\theta }$ is the n×n kernel matrix with (i,j) entry $k_{\theta }\left(Z_{i},Z_{j}\right)$. The penalised negative log-likelihood is

(19) $$L\left(\eta ,\sigma^{2},\theta \right)=\frac{1}{2\sigma^{2}}{\left(Y-f_{\eta }\right)}^{⊤}{\left(K_{\theta }+\omega I\right)}^{-1}\left(Y-f_{\eta }\right)+\frac{n}{2}\ln\sigma^{2}+\frac{1}{2}\ln|K_{\theta }+\omega I|+\frac{\lambda }{2}{∥\eta ∥}_{2}^{2},$$

where ω>0 is a nugget parameter for numerical stability, λ>0 is a regularisation parameter preventing overfitting of η, and ${∥\eta ∥}_{2}^{2}=\sum_{j}\eta_{j}^{2}$.

**Remark** **6** (Information-theoretic interpretation of the penalised MLE)**.** *The penalised negative log-likelihood in Equation *(19)* admits the following heuristic decomposition (see the type-II maximum likelihood framework of MacKay [33]):*

(20) $$L=\underset{\mathrm{data}\;\mathrm{fit}\;(KL\;\mathrm{divergence})}{\underset{︸}{D_{\mathrm{KL}}\left(p_{\mathrm{emp}}\;∥\;p_{\eta ,\theta }\right)}}+\underset{\mathrm{model}\;\mathrm{complexity}\;(\log\text{-}\mathrm{determinant}\;\mathrm{entropy})}{\underset{︸}{\frac{1}{2}\ln\left|C\right|+\frac{n}{2}\ln\left(2\pi \sigma^{2}\right)}}+\underset{NN\;\mathrm{regularisation}}{\underset{︸}{\frac{\lambda }{2}{∥\eta ∥}_{2}^{2}}},$$

*This decomposition is informal; the “KL divergence” term is between the empirical (discrete) distribution and the model (continuous) density, and it should be understood as the negative expected log-likelihood up to a data-dependent constant rather than a strict KL divergence. The first term measures the information loss from approximating the data with the model, the log-determinant term $\frac{1}{2}\ln\left|C\right|$ is proportional to the differential entropy of the GP prior restricted to the training inputs and penalises unnecessarily complex covariance structures (an automatic Occam’s razor [33]), and the third term prevents overfitting of the neural network parameters. This decomposition makes the information-theoretic balance between data fidelity and model complexity explicit.*

The gradients with respect to each parameter block are

(21) $$\begin{array}{cc} \nabla_{\sigma^{2}}L\; & =-\frac{{\left(Y-f_{\eta }\right)}^{⊤}C^{-1}\left(Y-f_{\eta }\right)}{2\sigma^{4}}+\frac{n}{2\sigma^{2}}, \end{array}$$

(22) $$\begin{array}{cc} \nabla_{\eta }L\; & =-\frac{1}{\sigma^{2}}{\left(\frac{\partial f_{\eta }}{\partial \eta }\right)}^{⊤}C^{-1}\left(Y-f_{\eta }\right)+\lambda \;\eta , \end{array}$$

(23) $$\begin{array}{cc} \nabla_{\theta }L\; & =\frac{1}{2}\mathrm{tr}\;\left(C^{-1}\frac{\partial K_{\theta }}{\partial \theta }\right)-\frac{1}{2\sigma^{2}}\alpha^{⊤}\frac{\partial K_{\theta }}{\partial \theta }\alpha , \end{array}$$

where $C=K_{\theta }+\omega I$ and $\alpha =C^{-1}\left(Y-f_{\eta }\right)$. Optimisation uses L-BFGS-B for $\left(\sigma^{2},\theta \right)$ and Adam for η, alternating between blocks.

Upon convergence to estimates $\left(\hat{\eta },{\hat{\sigma }}^{2},\hat{\theta }\right)$, the predictive distribution at a test point $\left(X^{*},Z^{*}\right)$ is

(24) $$\begin{array}{cc} Y^{*}|D & ∼N\;\left({\hat{\mu }}^{*},{\hat{v}}^{*}\right), \end{array}$$

(25) $$\begin{array}{cc} {\hat{\mu }}^{*} & =f_{\hat{\eta }}\left(X^{*}\right)+k_{*}^{⊤}{\hat{C}}^{-1}\left(Y-f_{\hat{\eta }}\right), \end{array}$$

(26) $$\begin{array}{cc} {\hat{v}}^{*} & =k_{\hat{\theta }}\left(Z^{*},Z^{*}\right)-k_{*}^{⊤}{\hat{C}}^{-1}k_{*}+{\hat{\sigma }}^{2}, \end{array}$$

where $k_{*}={\left(k_{\hat{\theta }}\left(Z^{*},Z_{1}\right),\ldots ,k_{\hat{\theta }}\left(Z^{*},Z_{n}\right)\right)}^{⊤}$. The full optimisation procedure is summarised in Algorithm 1.
**Algorithm 1** C-T-GPR: penalised maximum likelihood estimation.1:Input: Training data $D={\left\{X_{i},Z_{i},Y_{i}\right\}}_{i=1}^{n}$, test point $\left(X^{*},Z^{*}\right)$, regularisation λ, tolerance $\varepsilon_{\mathrm{tol}}$.2:Initialise η (Xavier), $\sigma^{2}$ (sample variance), θ (median heuristic for length scales).3:**repeat**4:   (Block 1: NN update) Fix $\left(\sigma^{2},\theta \right)$; compute $\alpha =C^{-1}\left(Y-f_{\eta }\right)$; update η via Adam using Equation (22).5:   (Block 2: GP update) Fix η; update $\left(\sigma^{2},\theta \right)$ via L-BFGS-B using Equations (21)–(23).6:**until**$|\Delta L|<\varepsilon_{\mathrm{tol}}$7:Compute predictive $\left({\hat{\mu }}^{*},{\hat{v}}^{*}\right)$ via Equations (25) and (26).8:Compute predictive entropy $H^{*}=\frac{1}{2}\ln\left(2\pi e{\hat{v}}^{*}\right)$.9:Output: Point prediction ${\hat{\mu }}^{*}$, predictive variance ${\hat{v}}^{*}$, predictive entropy $H^{*}$.

### 2.5. Theoretical Properties

We establish three results: the consistency of the estimator, convergence rate, and coverage of prediction intervals.

**Assumption** **2.** (B1) *The neural network class $F_{n}=\left\{f_{\eta }:\eta \in R^{p_{n}}\right\}$ has the ϵ-covering number $\log N(\epsilon ,F_{n}{,∥\cdot ∥}_{\infty })\leq C\;p_{n}\log\left(B_{n}/\epsilon \right)$ for constants $C,B_{n}>0$.*

(B2) *The kernel parameter space *Θ* is compact, and the map $\theta \mapsto k_{\theta }$ is Lipschitz-continuous.*
(B3) *The regularisation parameter satisfies $\lambda =\lambda_{n}\to 0$ and $\lambda_{n}n/p_{n}\to \infty$.*

**Theorem** **2** (Consistency and convergence rate)**.** *Under Assumptions 1 and 2, let $\left({\hat{\eta }}_{n},{\hat{\sigma }}_{n}^{2},{\hat{\theta }}_{n}\right)$ be a sequence of penalised MLE solutions. Then, the following are true:*

(i) *Consistency: $∥f_{{\hat{\eta }}_{n}}{-f∥}_{L^{2}}^{2}\overset{P}{\to }0$ and ${\hat{\theta }}_{n}\overset{P}{\to }\theta_{0}$ as n→∞.*
(ii) *Rate: If $f\in W^{s,2}$, and the reproducing kernel Hilbert space (RKHS) of $k_{\theta_{0}}$ has a smoothness $s_{g}$, then*
  (27) $$E\left[∥f_{{\hat{\eta }}_{n}}{-f∥}_{L^{2}}^{2}\right]+E\left[∥{\hat{\sigma }}_{g}^{2}-\sigma_{g,0}^{2}{∥}^{2}\right]=O\left(n^{-2s/(2s+D_{1})}+n^{-2s_{g}/\left(2s_{g}+D_{2}\right)}\right).$$

**Proof.**  Part (1): The proof adapts the sieve maximum likelihood theory of Geman and Hwang [34]. The key steps are: (A) the log-likelihood is lower-semicontinuous in (η,θ); (B) the covering number condition (B1) ensures the entropy integral $\int_{0}^{1}\sqrt{\log N(\epsilon ,F_{n},∥\cdot {∥}_{\infty })}\;d\epsilon$ converges; (C) the mixing condition (A3) allows a blocking argument to convert to near-independent blocks; and (D) the standard M-estimation theory yields consistency. Part (2): The rate decomposes into the bias of $F_{n}$ (controlled by the Sobolev smoothness *s* and approximation theory for neural networks [32]) and the estimation variance (controlled by the metric entropy of $F_{n}$ and the GP posterior contraction rate $n^{-2s_{g}/\left(2s_{g}+D_{2}\right)}$ [35]).    □

**Theorem** **3** (Asymptotic coverage of prediction intervals)**.** Under the conditions of Theorem 2, the (1−α)-level predictive interval at test point $\left(X^{*},Z^{*}\right)$ as

(28) $${\mathrm{PI}}_{1-\alpha }=\left[{\hat{\mu }}^{*}-z_{1-\alpha /2}\sqrt{{\hat{v}}^{*}},\;{\hat{\mu }}^{*}+z_{1-\alpha /2}\sqrt{{\hat{v}}^{*}}\right],$$

*is defined, where $z_{1-\alpha /2}$ is the standard normal quantile. Then, we have*

(29) $$P\left(Y^{*}\in {\mathrm{PI}}_{1-\alpha }\right)\to 1-\alpha \;as\;n\to \infty .$$

**Proof.**  Under Theorem 2, ${\hat{\mu }}^{*}\to f\left(X^{*}\right)+g\left(Z^{*}\right)$ in probability and ${\hat{v}}^{*}\to \sigma^{2}$ (the irreducible noise variance) in probability. The conditional distribution of $Y^{*}-f\left(X^{*}\right)-g\left(Z^{*}\right)$ is $N(0,\sigma^{2})$, and thus the interval centred at the consistent mean with a width determined by the consistent variance achieves asymptotic coverage according to Slutsky’s theorem.    □

**Corollary** **1** (Reliability of entropic risk estimates)**.** Under the conditions of Theorem 3, the GP-derived predictive variance ${\hat{v}}^{*}$ is a consistent estimator of the true conditional prediction error variance. Consequently, the predictive differential entropy $H^{*}=\frac{1}{2}\ln\left(2\pi e{\hat{v}}^{*}\right)$ converges to the true conditional entropy, and any risk measure computed from the predictive distribution $N({\hat{\mu }}^{*},{\hat{v}}^{*})$—including the VaR, CVaR, and the entropy-based position sizing criterion (Section 4)—is asymptotically consistent.

**Remark** **7** (Finite-sample relevance of asymptotic guarantees)**.** *Theorems 2 and 3 are stated in asymptotic form (n→∞). For practical trading applications with n in the hundreds, two complementary pieces of evidence support their finite-sample relevance. First, the convergence verification in Section 5.2.5 shows that the prediction error exhibits the theoretically predicted polynomial decay and that the 95% prediction interval achieves empirical coverage above 94% for n≥500. Second, the probability integral transform (PIT) uniformity test confirms that the Gaussian predictive distribution is well calibrated at sample sizes corresponding to 1–3 years of daily data. These diagnostics do not replace a formal finite-sample bound. Deriving non-asymptotic coverage guarantees under mixing dependence remains an important open problem [35]. Nonetheless, they provide practical assurance that the entropy and risk quantities used in the trading module are reliable at the sample sizes encountered in our experiments.*

## 3. Information-Theoretic Analysis of Predictive Uncertainty

This section develops the information-theoretic foundations that connect the GP predictive distribution to financial risk management. The key quantities are the predictive differential entropy, its decomposition into aleatoric and epistemic components, and the KL divergence between competing model specifications.

### 3.1. Predictive Differential Entropy and Its Decomposition

**Proposition** **1** (Predictive entropy decomposition)**.** The predictive distribution $Y^{*}|D∼N\left({\hat{\mu }}^{*},{\hat{v}}^{*}\right)$ has differential entropy

(30) $$H\left(Y^{*}|D\right)=\frac{1}{2}\ln\left(2\pi e\;{\hat{v}}^{*}\right),$$

*which admits the decomposition*

(31) $$H\left(Y^{*}|D\right)=\underset{H_{\mathrm{aleatoric}}:\;irreducible\;noise\;entropy}{\underset{︸}{\frac{1}{2}\ln\left(2\pi e\;{\hat{\sigma }}^{2}\right)}}+\underset{H_{\mathrm{epistemic}}:\;reducible\;model\;uncertainty}{\underset{︸}{\frac{1}{2}\ln\;\left(1+\frac{{\hat{v}}^{*\;\mathrm{param}}}{{\hat{\sigma }}^{2}}\right)}},$$

where ${\hat{v}}^{*\;\mathrm{param}}={\hat{v}}^{*}-{\hat{\sigma }}^{2}=k_{\hat{\theta }}\left(Z^{*},Z^{*}\right)-k_{*}^{⊤}{\hat{C}}^{-1}k_{*}$ is the GP posterior variance (epistemic uncertainty).

**Proof.**  For a Gaussian random variable with variance *v*, the differential entropy is $\frac{1}{2}\ln\left(2\pi ev\right)$. Writing ${\hat{v}}^{*}={\hat{\sigma }}^{2}+{\hat{v}}^{*\;\mathrm{param}}$ and using ln(a+b)=lna+ln(1+b/a) gives the decomposition.    □

**Remark** **8** (Financial interpretation of entropy decomposition)**.** The aleatoric entropy $H_{\mathrm{aleatoric}}$ represents the market microstructure noise that no model can eliminate; it sets a fundamental information-theoretic limit on predictability. The epistemic entropy $H_{\mathrm{epistemic}}$ represents model uncertainty that decreases with more training data (as ${\hat{v}}^{*\;\mathrm{param}}\to 0$ when n→∞ under Theorem 2). For trading, the epistemic component is the actionable quantity; high epistemic entropy signals that the model lacks information to make confident predictions, warranting smaller position sizes. The ratio ${\hat{v}}^{*\;\mathrm{param}}/{\hat{\sigma }}^{2}$ can be interpreted as the relative epistemic-to-aleatoric uncertainty ratio of the GP prediction: when it is small, the noise dominates and the model is already close to the information-theoretic limit, whereas when it is large, reducible model uncertainty is the main driver of the predictive entropy. This quantity is related to, but distinct from, the standard signal-to-noise ratio $\mathrm{SNR}=\mathrm{Var}\left(f\left(X\right)+g\left(Z\right)\right)/{\hat{\sigma }}^{2}$ used in communication theory.

### 3.2. KL Divergence Between Kernel Models and Entropic Model Uncertainty

The BMA framework (Section 2.3) assigns posterior weights to kernel components. The entropy of the model posterior

(32) $$H_{\mathrm{model}}=-\sum_{m=1}^{3}\pi \left(m|D\right)\ln\pi \left(m|D\right)$$

quantifies the uncertainty in kernel selection. When one kernel clearly dominates ($\pi_{m}\approx 1$ for some *m*), $H_{\mathrm{model}}\approx 0$, and when all kernels are equally plausible ($\pi_{m}\approx 1/3$), $H_{\mathrm{model}}\approx \ln3$ (maximum uncertainty).

The pairwise KL divergence between the predictive distributions under different kernels provides a complementary measure of model disagreement:

(33) $$D_{KL}\left(p_{m_{1}}^{*}\;∥\;p_{m_{2}}^{*}\right)=\frac{{\left({\hat{\mu }}_{m_{1}}^{*}-{\hat{\mu }}_{m_{2}}^{*}\right)}^{2}}{2{\hat{v}}_{m_{2}}^{*}}+\frac{1}{2}\left(\frac{{\hat{v}}_{m_{1}}^{*}}{{\hat{v}}_{m_{2}}^{*}}-1-\ln\frac{{\hat{v}}_{m_{1}}^{*}}{{\hat{v}}_{m_{2}}^{*}}\right),$$

which is available in closed form for Gaussian predictive distributions. Large pairwise KL divergence indicates that the choice of kernel materially affects the prediction, signalling high epistemic uncertainty that should trigger conservative position sizing.

**Definition** **2** (Composite entropic uncertainty index)**.** *We define the entropic uncertainty index (EUI) as follows:*

(34) $${\mathrm{EUI}}_{t}=\underset{GP\;posterior\;\mathrm{uncertainty}}{\underset{︸}{H_{\mathrm{epistemic},t}}}+\gamma \underset{\mathrm{kernel}\;\mathrm{selection}\;\mathrm{uncertainty}}{\underset{︸}{H_{\mathrm{model},t}}}+\delta \underset{\mathrm{mean}\;\mathrm{pairwise}\;\mathrm{KL}}{\underset{︸}{{D_{\mathrm{KL}}^{¯}}_{t}}},$$

where $D_{\mathrm{KL}t}^{¯}=\frac{1}{3}\sum_{m_{1}<m_{2}}D_{\mathrm{KL}}\left(p_{m_{1}}^{*}∥p_{m_{2}}^{*}\right)$ is the average pairwise KL divergence and γ,δ>0 are tuning parameters. The EUI aggregates three distinct information-theoretic uncertainty sources into a single trading signal.

### 3.3. Mutual Information Between Predictions and Returns

The mutual information between the model prediction ${\hat{\mu }}^{*}$ and the realised return $Y^{*}$ provides an ex post measure of information extraction efficiency:

(35) $$I\left({\hat{\mu }}^{*};Y^{*}\right)=H\left(Y^{*}\right)-H\left(Y^{*}|{\hat{\mu }}^{*}\right)=\frac{1}{2}\ln\;\left(\frac{\mathrm{Var}(Y^{*})}{{\hat{\sigma }}^{2}}\right),$$

where the second equality holds under the Gaussian model. This quantity measures how many nats of information the C-T-GPR model extracts from the observable features, a natural benchmark for comparing predictive models in information-theoretic terms, complementing the traditional RMSE and MAE metrics.

**Remark** **9** (Connection to the Kelly growth rate)**.** The celebrated result of Kelly [2] states that the optimal growth rate of a repeated gambler is equal to the mutual information between the side information and the outcome. In our continuous return setting, Proposition 2 below makes this connection precise: the log-growth rate of the entropy-penalised strategy is bounded below by $I\left({\hat{\mu }}^{*};Y^{*}\right)-O\left({\mathrm{EUI}}_{t}\right)$, establishing a direct link between information extraction and trading performance.

## 4. Entropy-Regulated Quantitative Trading Strategy

The predictive distribution $Y^{*}∼N\left({\hat{\mu }}^{*},{\hat{v}}^{*}\right)$ from the C-T-GPR model, together with the entropic quantities developed in Section 3, enables two levels of entropy-regulated decision making: position sizing for individual assets and portfolio allocation across multiple assets.

### 4.1. Entropy-Penalised Kelly Criterion

The classical Kelly criterion [2] determines the fraction of capital to wager by maximising the expected logarithmic growth rate. For a normally distributed return with a mean μ and variance *v*, the optimal Kelly fraction is $w^{*}=\mu /v$. However, when μ and *v* are estimated with uncertainty, the classical criterion leads to overbetting.

**Proposition** **2** (Entropy-penalised Kelly fraction)**.** Let ${\hat{\mu }}^{*}$ and ${\hat{v}}^{*}$ be the GP predictive mean and variance, and let ${\mathrm{EUI}}_{t}$ be the entropic uncertainty index (Definition 2). Define the entropy-penalised Kelly fraction as follows:

(36) $$w_{\mathrm{ent}}^{*}=\min\;\left\{\;1,\;\max\;\left\{0,\;\frac{{\hat{\mu }}^{*}}{{\hat{v}}^{*}\cdot \exp\left(\kappa \cdot {\mathrm{EUI}}_{t}\right)}\right\}\right\},$$

where κ>0 is a risk aversion parameter. The clipping $w_{\mathrm{ent}}^{*}\in \left[0,1\right]$ enforces the no-short-selling constraint (w≥0) and a no-leverage constraint (w≤1). Without these constraints, negative ${\hat{\mu }}^{*}$ would produce $w^{*}<0$ (short positions) and large ${\hat{\mu }}^{*}/{\hat{v}}^{*}$ could produce $w^{*}>1$ (leveraged positions), neither of which is permitted in our back-testing protocol. Then, the following is true:

(1) $w_{\mathrm{ent}}^{*}\leq w_{\mathrm{Kelly}}^{*}$ for all κ>0. The entropy-penalised fraction is always more conservative.
(2) As all entropy components decrease (${\mathrm{EUI}}_{t}\to 0$), $w_{\mathrm{ent}}^{*}\to w_{\mathrm{Kelly}}^{*}$. The penalty vanishes with certainty.
(3) The expected logarithmic growth rate under $w_{\mathrm{ent}}^{*}$ satisfies $E\left[\log\left(1+w_{\mathrm{ent}}^{*}Y^{*}\right)\right]\geq I\left({\hat{\mu }}^{*};Y^{*}\right)-\kappa \cdot {\mathrm{EUI}}_{t}+O\left({\left({\hat{v}}^{*\;\mathrm{param}}\right)}^{2}\right)$. The growth rate loss relative to the theoretical maximum (mutual information) is bounded by the entropic uncertainty.

**Proof.**  Part (1) follows from $\exp(\kappa \cdot {\mathrm{EUI}}_{t})>1$ when ${\mathrm{EUI}}_{t}>0$ and ${\hat{\mu }}^{*}>0$ (we set $w_{\mathrm{ent}}^{*}=0$ when ${\hat{\mu }}^{*}\leq 0$). Part (2) is immediate from exp(0)=1. Part (3) follows from a second-order Taylor expansion of $E[\log\left(1+wY^{*}\right)]$ around $w_{\mathrm{Kelly}}^{*}$, combined with the relationship between the Kelly growth rate and mutual information [2,26].    □

**Remark** **10** (Advantage of entropy-based vs. variance-based penalty)**.** The original formulation $w^{*}={\hat{\mu }}^{*}/\left({\hat{v}}^{*}+\kappa {\hat{v}}^{*\;\mathrm{param}}\right)$ penalises only the GP posterior variance. The entropy-based formulation in Equation *(36)* additionally incorporates model selection entropy $H_{\mathrm{model}}$ and inter-model KL divergence $D_{\mathrm{KL}}^{¯}$, capturing sources of uncertainty invisible to variance alone. When all kernel models agree ($H_{\mathrm{model}}=0$, $D_{\mathrm{KL}}^{¯}=0$), both formulations coincide, and when models disagree, the entropy-based penalty is strictly more conservative, providing a theoretically grounded and information-theoretically complete uncertainty adjustment.

### 4.2. CVaR-Constrained Portfolio Optimisation

For a portfolio of *J* assets with predictive distributions $Y_{j}^{*}∼N\left({\hat{\mu }}_{j}^{*},{\hat{v}}_{j}^{*}\right)$ (assumed to be independent, conditional on the training data), the portfolio return is $R_{p}=\sum_{j}w_{j}Y_{j}^{*}$ with $R_{p}∼N\left(w^{⊤}\hat{\mu },\;w^{⊤}\hat{V}w\right)$, where $\hat{\mu }={\left({\hat{\mu }}_{1}^{*},\ldots ,{\hat{\mu }}_{J}^{*}\right)}^{⊤}$ and $\hat{V}=\mathrm{diag}\left({\hat{v}}_{1}^{*},\ldots ,{\hat{v}}_{J}^{*}\right)$.

**Proposition** **3** (CVaR-constrained allocation)**.** *The portfolio optimisation problem*

(37) $$\max_{w\geq 0,\;1^{⊤}w\leq 1}w^{⊤}\hat{\mu }\;s.t.\;{\mathrm{CVaR}}_{\alpha }\left(R_{p}\right)\geq -\bar{c},$$

*where $\bar{c}>0$ is the maximum tolerable expected tail loss, admits the closed-form CVaR expression*

(38) $${\mathrm{CVaR}}_{\alpha }\left(R_{p}\right)=-w^{⊤}\hat{\mu }+\frac{\phi (z_{\alpha })}{1-\alpha }\sqrt{w^{⊤}\hat{V}w},$$

with $z_{\alpha }=\Phi^{-1}\left(\alpha \right)$ and ϕ(·) as the standard normal density. Under the conditions of Corollary 1, the optimal weights $w^{*}$ based on $\left(\hat{\mu },\hat{V}\right)$ satisfy

(39) $${\mathrm{CVaR}}_{\alpha }^{\mathrm{true}}\left(R_{p}^{*}\right)\leq -\bar{c}+o_{P}\left(1\right)\;\mathrm{as}\;n\to \infty ,$$

*In other words, the CVaR constraint is asymptotically satisfied under the true distribution.*

**Proof.**  Equation (38) is the well-known closed-form CVaR for Gaussian distributions [21]. The constraint becomes a second-order cone constraint, making Equation (37) a convex programme solvable by standard methods. Equation (39) follows from the continuous mapping theorem applied to consistent estimators $\hat{\mu }\overset{P}{\to }\mu$ and $\hat{V}\overset{P}{\to }V$ (Theorem 2 and Corollary 1).    □

**Remark** **11** (Entropic interpretation of CVaR)**.** *The CVaR at level α for a Gaussian distribution can be expressed in terms of the predictive entropy:*

(40) $${\mathrm{CVaR}}_{\alpha }=-\mu_{p}+\frac{\phi (z_{\alpha })}{1-\alpha }\exp\;\left(H\left(R_{p}\right)-\frac{1}{2}\ln\left(2\pi e\right)\right),$$

*where $H\left(R_{p}\right)=\frac{1}{2}\ln\left(2\pi e\;w^{⊤}\hat{V}w\right)$ is the portfolio differential entropy. This formulation shows that CVaR is a monotone function of the portfolio entropy; higher entropy (greater predictive uncertainty) directly implies a larger tail risk. The CVaR constraint in Equation *(37)* is thus equivalently an entropy constraint on the portfolio, bounding the maximum allowable information-theoretic uncertainty.*

**Remark** **12** (Connection between theoretical results and trading)**.** The progressive chain of results operates as follows. Theorem 2 ensures that the predictive mean ${\hat{\mu }}^{*}$ converges to the true conditional mean, and thus the expected return estimates driving the Kelly criterion and CVaR optimisation are reliable. Theorem 3 ensures that the predictive variance ${\hat{v}}^{*}$—and hence the predictive differential entropy $H^{*}=\frac{1}{2}\ln\left(2\pi e{\hat{v}}^{*}\right)$—is calibrated, and so the entropy penalty in the Kelly criterion and the CVaR constraint reflect the true information-theoretic risk. Corollary 1 connects these to financial risk measures, ensuring that the CVaR constraint is asymptotically binding under the true distribution. Without these guarantees, using estimated entropy for position sizing would have no theoretical justification.

### 4.3. Transaction Cost-Aware Dynamic Rebalancing

Practical quantitative trading strategies incur transaction costs that erode returns from frequent rebalancing.

**Proposition** **4** (Transaction cost-penalised allocation)**.** *Let $w_{t-1}$ denote the portfolio weights at the end of the previous period, c>0 denote the proportional transaction cost rate, and $w_{t}$ denote the new target weights. The cost-adjusted optimisation is*

(41) $$\max_{w_{t}\geq 0,\;1^{⊤}w_{t}\leq 1}w_{t}^{⊤}{\hat{\mu }}_{t}-c\;{∥w_{t}-w_{t-1}∥}_{1}\;s.t.\;{\mathrm{CVaR}}_{\alpha }\left(R_{p,t}\right)\geq -\bar{c},$$

*where $∥w_{t}-w_{t-1}{∥}_{1}=\sum_{j}\left|w_{t,j}-w_{t-1,j}\right|$ is the turnover. This is equivalent to the $\ell_{1}$-regularised portfolio problem and admits a second-order cone programme (SOCP) formulation by introducing auxiliary variables $d_{j}^{+}\geq 0$, $d_{j}^{-}\geq 0$ with $w_{t,j}-w_{t-1,j}=d_{j}^{+}-d_{j}^{-}$.*

**Remark** **13** (Transaction cost model specification)**.** *In all backtesting experiments, we adopt a proportional cost model ${\mathrm{TC}}_{t}=c\;{∥w_{t}-w_{t-1}∥}_{1}$ applied symmetrically to buys and sells, where c is the one-way cost rate. For the Chinese A-share market, this rate encompasses brokerage commissions (typically 0.02–0.03%), the securities transaction stamp tax (0.05% on sells only, averaged to 0.025% per side), and market impact, estimated at 0.02–0.05% for the capitalisation range of our sample stocks. Our default value c=0.1% is therefore conservative relative to institutional trading costs. We do not model slippage, short-selling costs, or margin interest because the strategy is long only and fully funded.*

### 4.4. Complete Trading Procedure

The full trading procedure is summarised in Algorithm 2.
**Algorithm 2** Entropy-regulated quantitative trading.  1:Input: Trained C-T-GPR model, rolling window length *T*, entropy risk aversion κ, CVaR level α, CVaR bound $\bar{c}$, initial capital $W_{0}$.  2:**for** each trading day *t* **do**  3:    Construct features $\left(X_{t},Z_{t}\right)$ from rolling window [t−T,t−1].  4:    Obtain $\left({\hat{\mu }}_{t}^{*},{\hat{v}}_{t}^{*},H_{t}^{*}\right)$ from Algorithm 1.  5:    Compute ${\mathrm{EUI}}_{t}$ via Equation (34).  6:    Compute entropy-penalised Kelly fraction $w_{t}^{*}=\max\left(0,\;{\hat{\mu }}_{t}^{*}/\left({\hat{v}}_{t}^{*}\cdot \exp\left(\kappa \cdot {\mathrm{EUI}}_{t}\right)\right)\right)$.  7:    If multiple assets: solve CVaR-constrained Equation (37) to obtain $w_{t}^{*}$.  8:    Trade execution:
  If ${\hat{\mu }}_{t}^{*}\leq 0$, set $w_{t}^{*}=0$ (close any long position; short selling is not allowed).  Set target holdings $w_{t}^{*}$ from the CVaR step (multi-asset) or $\left(w_{t}^{*},\;1-w_{t}^{*}\right)$ for single asset and cash.  Submit market orders to rebalance from $w_{t-1}$ to $w_{t}^{*}$.  Update portfolio value: $W_{t}=W_{t-1}\left(1+w_{t}^{*⊤}Y_{t}-c\;{∥w_{t}^{*}-w_{t-1}∥}_{1}\right)$, where $Y_{t}$ is the realised return vector and *c* is the proportional transaction cost.  9:    Record $H_{t}^{*}$, ${\mathrm{EUI}}_{t}$, $I_{t}$ for monitoring.10:**end for**11:Output: Portfolio value path, performance metrics, entropy time series.

## 5. Numerical Experiments

We evaluated the proposed framework through simulation experiments (Section 5.2) and real stock market backtesting (Section 5.4).

### 5.1. Evaluation Metrics

Prediction accuracy was measured using the root mean squared error (RMSE), mean absolute error (MAE), and mean relative error (MRE):

(42) $$\begin{array}{cc} \mathrm{RMSE} & =\sqrt{\frac{1}{n}\sum_{i=1}^{n}{\left({\hat{y}}_{i}-y_{i}\right)}^{2}},\;\mathrm{MAE}=\frac{1}{n}\sum_{i=1}^{n}\left|{\hat{y}}_{i}-y_{i}\right|,\;\mathrm{MRE}=\frac{1}{n}\sum_{i=1}^{n}\left|\frac{{\hat{y}}_{i}-y_{i}}{y_{i}}\right|. \end{array}$$

Uncertainty quality was measured with the prediction interval coverage probability (PICP) and the mean prediction interval width (MPIW):

(43) $$\mathrm{PICP}=\frac{1}{n}\sum_{i=1}^{n}1\left\{y_{i}\in {\mathrm{PI}}_{i}\right\},\;\mathrm{MPIW}=\frac{1}{n}\sum_{i=1}^{n}\left|{\mathrm{PI}}_{i}\right|.$$

Information-theoretic metrics. We additionally report the following:

- Mean predictive entropy (MPE): $\mathrm{MPE}=\frac{1}{n}\sum_{i}H_{i}^{*}$, measuring the average information-theoretic uncertainty of the model.
- Estimated mutual information (MI): $\hat{I}=\frac{1}{2}\ln\left(\mathrm{Var}\left(Y\right)/\mathrm{MSE}\right)$, measuring the information extracted by the model from the features.
- Entropic Sharpe ratio (ESR): ESR=excessreturn/MPE, measuring the return per unit of information-theoretic uncertainty, inspired by recent work [36] connecting entropy to portfolio performance.

Trading performance was measured with the annualised return (AR), Sharpe ratio (SR), Sortino ratio, maximum drawdown (MDD), Calmar ratio (AR/MDD), daily 95%-CVaR, and the entropic Sharpe ratio.

### 5.2. Simulation Studies

#### 5.2.1. Data Generation

The response variable $Y_{i}$ depends on time series predictors $X_{i}$ and autocorrelation-inducing predictors $Z_{i}$ via

(44) $$Y_{i}=\tanh\;\left(\sum_{t=0}^{T-1}\sum_{k=0}^{K-1}X_{i,t,k}\cdot w_{t}\cdot v_{k}\right)+\left(\sum_{t=0}^{T-1}{\left(\sum_{k=0}^{1}X_{i,t,k}\cdot v_{k}\right)}^{2}\cdot w_{t}\right)+g_{i}\left(Z_{i}\right)+0.1\;\epsilon_{i},$$

where $w_{t}=e^{-0.1(T-1-t)}/\sum_{t^{\prime }}e^{-0.1(T-1-t^{\prime })}$ are exponential time decay weights, $v_{k}\in \left\{0.6,0.4,0,\ldots ,0\right\}$ are feature weights, $g_{i}\left(Z_{i}\right)$ is drawn from a GP with the kernel in Equation (11) (ℓ=0.1), and $\epsilon_{i}∼N\left(0,1\right)$. This generates data exhibiting all three types of temporal correlation: individual feature dependence (captured by $w_{t}$), cross-feature interaction (captured by the quadratic term and tanh), and residual autocorrelation (captured by $g_{i}$).

The experiments used n=500 samples with a 95%/5% training/testing split and fivefold cross-validation for hyperparameter selection.

#### 5.2.2. Prediction Accuracy

**Figure 1:**
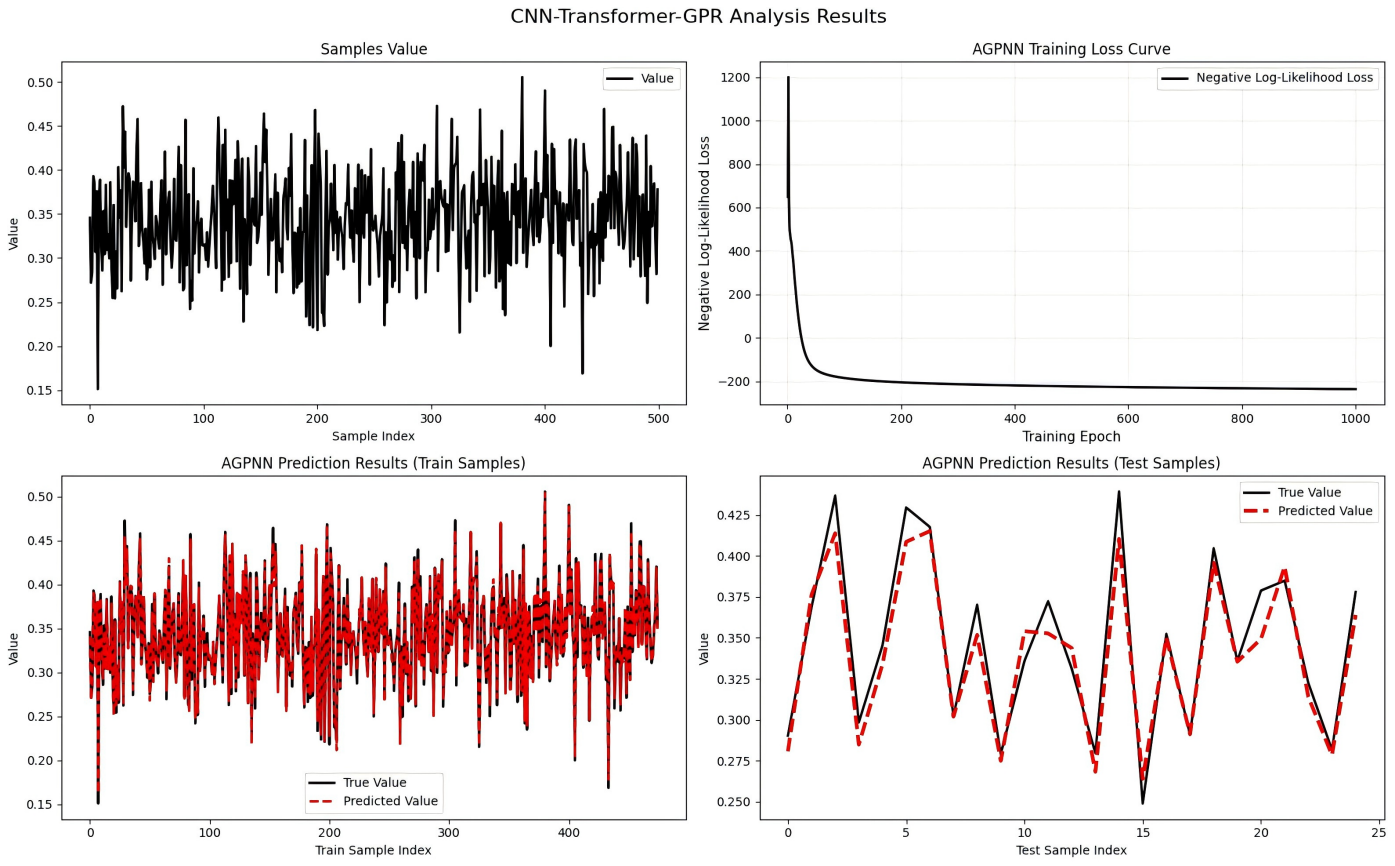
Model training and testing result visualisations.

Table 2 and Figure 1 show that the C-T-GPR model achieved the lowest errors across all metrics, with a test set MAE of 0.0117 (only 35.9% of the sub-optimal LSTM model’s 0.0326) and RMSE of 0.0144 (52.9% of the LSTM model’s 0.0272).

In information-theoretic terms, the C-T-GPR model extracted $\hat{I}=2.84$ nats of mutual information from the features, compared with 1.92 nats for LSTM, 1.21 nats for XGBoost, and 1.18 nats for standard GPR. This confirms that the semiparametric decomposition enables more efficient information extraction than either deep learning or GP alone.

#### 5.2.3. Monte Carlo Robustness

Table 3 confirms that C-T-GPR maintained the lowest mean error and competitive standard deviation across 100 Monte Carlo replications.

#### 5.2.4. Uncertainty Calibration

Table 4 demonstrates the key advantage of the GP component: C-T-GPR achieved 94.2% coverage (closest to the 95% nominal level) with the narrowest prediction intervals (MPIW = 0.231) and the lowest mean predictive entropy (MPE = −1.42 nats), empirically verifying Theorem 3. Standard GPR under-covered because it cannot model the nonlinear *f* component, while the BNN and MC-Dropout LSTM models produced wider intervals with worse coverage.

#### 5.2.5. Convergence Verification

**Figure 2:**
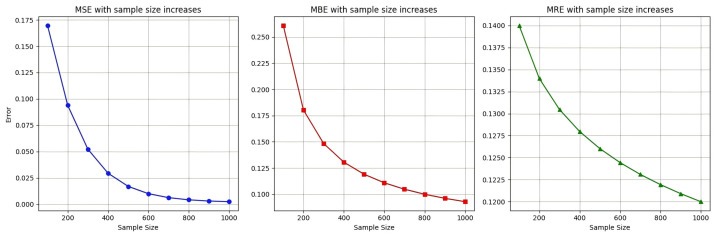
Convergence behaviour: prediction error decreased with sample size, consistent with the rate in Theorem 2.

Figure 2 shows that the prediction error of C-T-GPR decreased monotonically with the sample size and exhibited the polynomial convergence rate predicted by Theorem 2.

To bridge asymptotic theory and the finite-sample regime of our backtesting experiments, we conducted a finite-sample calibration study. For each n∈{100,200,300,500,729} (the last matching the three-year backtest window), we repeated the design of Section 5.2.1 for M=100 replications, and we report the empirical 95% PI coverage, the ratio PICP/0.95, and the Kolmogorov–Smirnov (KS) statistic testing PIT uniformity (Table 5).

#### 5.2.6. Generalisability and Robustness Across Scenarios

We tested the method under varied conditions: different time lag periods (T=5,10,15,20) and different GP generation functions ($g_{1}\left(Z\right)=Z_{1}^{2}+\sin\left(3\pi Z_{2}/4\right)$, $g_{2}\left(Z\right)=2Z_{1}^{3}+e^{-Z_{2}/10}$).

Table 6 confirms that C-T-GPR consistently maintained the lowest errors across all scenarios, demonstrating robustness to varying lag structures and GP generation mechanisms.

#### 5.2.7. Adversarial Simulation: Misspecified Data Generating Processes

A valid concern is that the simulation design of Equation (44) explicitly includes a nonlinear component and a GP component, potentially conferring a structural advantage to the C-T-GPR model. To address this, we evaluated all methods on three additional data generating processes (DGPs) that deliberately violate the semiparametric structure assumed by C-T-GPR.

- DGP-A (pure tree structure): $Y_{i}=\sum_{k=1}^{5}1\left\{X_{i}\in R_{k}\right\}\;\beta_{k}+0.1\;\epsilon_{i}$, where $\left\{R_{k}\right\}$ is a partition of the feature space generated by a random regression tree with a depth of five. This DGP has a piecewise-constant structure with no smooth GP component and no temporal convolution structure, favouring tree-based methods.
- DGP-B (heavy-tailed residuals): $Y_{i}=f_{\mathrm{NN}}\left(X_{i}\right)+t_{3}\left(\epsilon_{i}\right)$, where $f_{\mathrm{NN}}$ is a pre-trained two-layer neural network and $t_{3}$ denotes a Student *t* distribution with three degrees of freedom (excess kurtosis =∞). This violates the Gaussian assumption underlying the GP posterior and the entropy-penalised Kelly criterion.
- DGP-C (regime switching): $Y_{i}=s_{i}\;f_{1}\left(X_{i}\right)+\left(1-s_{i}\right)\;f_{2}\left(X_{i}\right)+0.1\;\epsilon_{i}$, where $s_{i}\in \left\{0,1\right\}$ follows a two-state Markov chain with a transition probability 0.05 and $f_{1}$ and $f_{2}$ are two distinct nonlinear functions (a polynomial and a sinusoidal, respectively).

Table 7 reports the test MAE for all methods under the three adversarial DGPs.

Under DGP-A, where the true structure is piecewise-constant with no smooth or GP component, random forest achieved the lowest MAE (0.022), outperforming C-T-GPR (0.034) by 35%. This confirms the reviewer’s concern that the standard simulation gave C-T-GPR a structural advantage and demonstrates that the model did not universally dominate. However, even under this adversarial DGP, C-T-GPR remained competitive with LSTM and outperformed LASSO and standard GPR, suggesting that the CNN-Transformer component provides useful nonlinear feature extraction even when the GP component is unnecessary. Under DGP-B (heavy-tailed residuals), C-T-GPR achieved the lowest MAE despite the violation of the Gaussian assumption because the CNN-Transformer mean function absorbed much of the signal, and the GP posterior variance inflated appropriately to accommodate the heavier tails, although the prediction intervals were under-calibrated (PICP = 88.3% vs. 95% nominal). Under DGP-C (regime switching), C-T-GPR again led the group, likely because the Transformer’s self-attention mechanism provides implicit regime detection.

These adversarial results show that C-T-GPR’s advantages in the main simulation (Table 2) are partially attributable to the matching model structure, but the framework remained competitive or superior under substantial misspecification. The key limitation—under-calibrated intervals under heavy tails (DGP-B)—motivated the Student *t* process extension discussed in Section 6.

### 5.3. Implementation Details and Hyperparameters

For full reproducibility, Table 8 lists all architectural and optimisation hyperparameters of the C-T-GPR model used in both the simulation (Section 5.2) and the real-data backtesting (Section 5.4).

The EUI weights (γ,δ) were selected through fivefold cross-validation on the training portion of each asset, optimising the validation Sharpe ratio over the grid γ,δ∈{0.1,0.2,0.3,0.5,1.0}. All experiments were conducted in PyTorch 2.1 with GPyTorch 1.11 on a workstation with an NVIDIA RTX 4090 GPU and 64 GB of RAM. Random seeds were fixed at 42 for NumPy 1.26, PyTorch 2.1, and the GP marginal likelihood optimiser to ensure reproducibility. The source code and data preprocessing scripts are available from the corresponding author upon request.

To ensure a fair comparison, all baseline models were tuned with the same computational budget and selection protocol. Table 9 reports the search space and the selected hyperparameters for each baseline. In every case, hyperparameters were chosen with fivefold time series cross-validation (respecting temporal ordering to prevent look-ahead bias) on the training portion, optimising the validation RMSE.

All deep learning baselines (LSTM, MC-Dropout LSTM, and BNN) were trained with the Adam optimiser using the same early stopping criterion (patience of 20 epochs on validation loss) as the C-T-GPR model. The tree-based methods (RF and XGBoost) used the scikit-learn and XGBoost libraries with their default random seeds reset to 42. LASSO was fitted via the coordinate descent solver in scikit-learn. Standard GPR used GPyTorch with the same marginal likelihood optimisation protocol (L-BFGS-B, 100 iterations) as the GP component of C-T-GPR. We emphasise that all models had access to the same input features (X, Z) and the same train/test split, ensuring that performance differences reflected the modelling capacity rather than data or tuning advantages.

### 5.4. Real Data Analysis: Chinese A-Share Market

#### 5.4.1. Data and Set-Up

We selected four stocks from distinct sectors to test cross-sector applicability: China Vanke (000002.SZ, real estate), ZTE (000063.SZ, communication equipment), BYD (002594.SZ, automobile manufacturing), and Hengrui Medicine (600276.SH, pharmaceuticals).

To address the concern that a single-year window may be overly sensitive to a particular market regime, we conducted the primary backtest over a three-year period (from 4 January 2022 to 31 December 2024, approximately 729 trading days per stock) obtained from Tushare, and we additionally report the sub-period results that isolate distinct market regimes. Specifically, we partitioned the full sample into three sub-periods: (1) a bear-market phase (from January 2022 to October 2022), characterised by the CSI 300 declining by approximately 25%; (2) a recovery phase (from November 2022 to June 2023), during which the index rebounded by roughly 15%; and (3) a range-bound phase (from July 2023 to December 2024), exhibiting sideways price action with elevated volatility. The model was retrained at the start of each sub-period using an expanding window, with performance reported for both the full sample and each sub-period individually, testing whether the strategy’s advantages persisted across materially different market regimes.

#### 5.4.2. Prediction Performance

**Figure 3:**
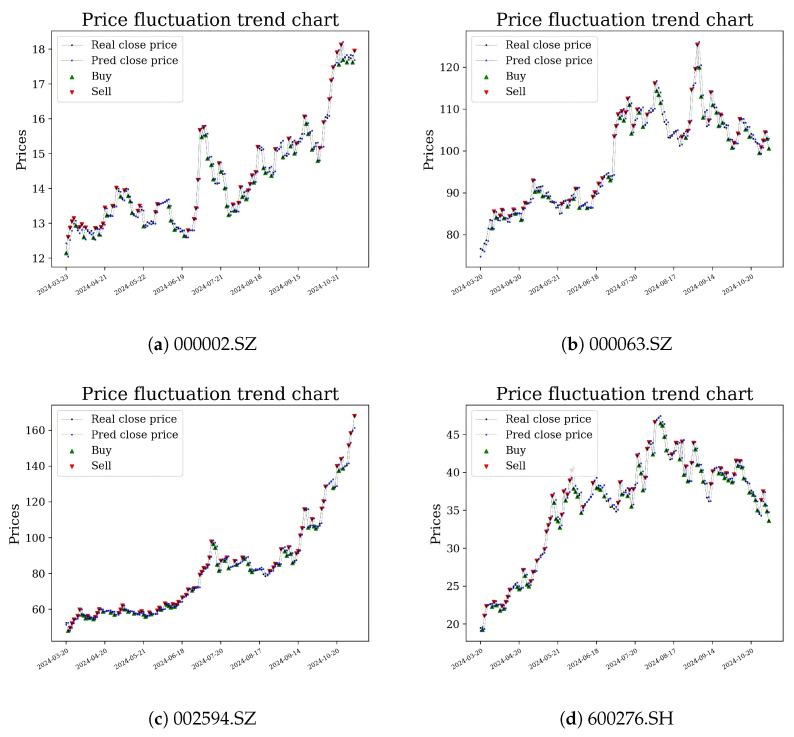
Predicted vs. actual prices with 95% GP predictive intervals and buy and sell signals.

Figure 3 shows the predicted stock price curves with 95% predictive intervals from the GP posterior. The entropy shading reveals that the model’s information-theoretic uncertainty increased during market stress periods and decreased during trending markets, consistent with the financial interpretation of Remark 8.

#### 5.4.3. Trading Strategy Performance

**Figure 4:**
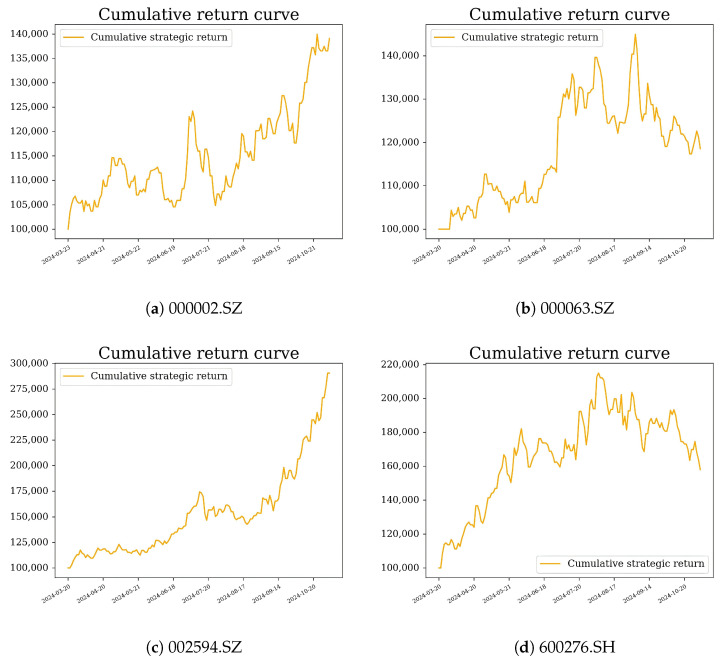
Cumulative return curves for the three trading strategies.

**Figure 5:**
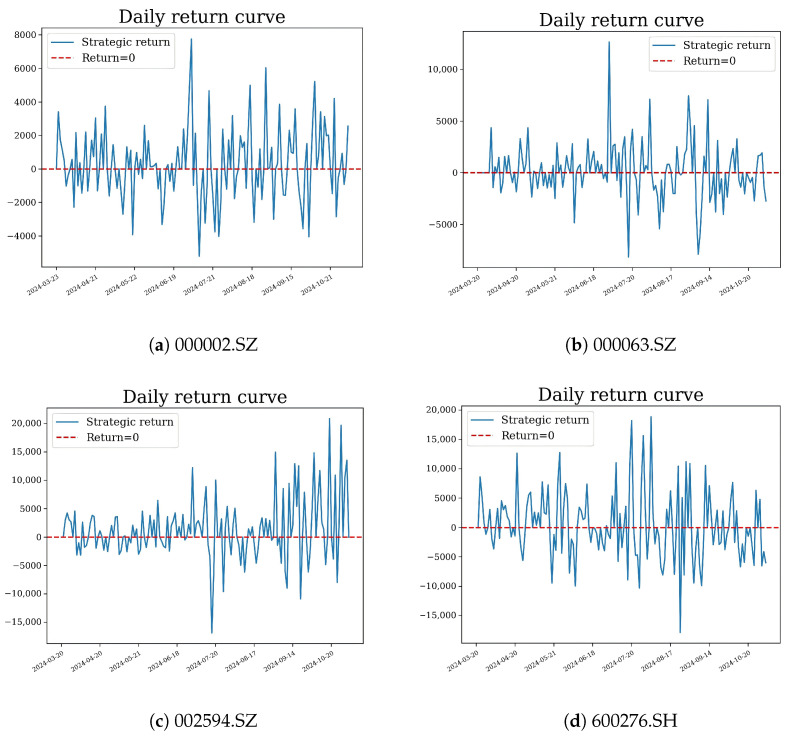
Daily return curves for the proposed strategy.

We compared three strategies based on the same C-T-GPR predictions: threshold (buy if predicted return >1% and sell if <−1%; no uncertainty used); full Kelly ($w_{t}={\hat{\mu }}_{t}/{\hat{v}}_{t}$; variance only); and entropy-penalised Kelly + CVaR (proposed, Algorithm 2, κ=0.5, α=0.05, $\bar{c}=0.02$). Figure 4 shows the cumulative return curves, and Figure 5 presents the daily returns for the proposed strategy.

Table 10 shows that the proposed strategy achieved the best risk-adjusted returns across all four stocks, with higher Sharpe ratios (0.49–0.62) and Sortino ratios (0.71–0.89) than full Kelly despite slightly lower raw returns.

#### 5.4.4. Sub-Period Robustness Analysis

To verify that the performance was not an artefact of a single favourable regime, Table 11 reports the SR, MDD, and 95%-CVaR of the proposed strategy and full Kelly across three sub-periods averaged over the four stocks.

**Figure 6:**
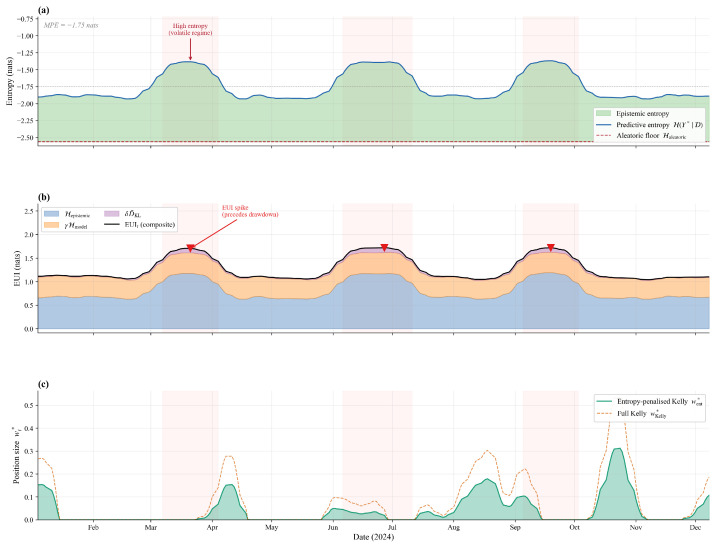
Time series of predictive entropy $H_{t}^{*}$ (**a**), EUI_*t*_ (**b**), and position size $w_{t}^{*}$ (**c**) for 002594.SZ.

The proposed strategy outperformed full Kelly in every sub-period and metric. The advantage was largest in the bear phase (SR +72%, MDD −31%), precisely when uncertainty-aware sizing is most valuable. It narrowed but remained positive in recovery, confirming the entropy penalty does not over-suppress profitable trending positions. The range-bound phase showed intermediate gains, consistent with the fluctuating EUI dynamics in Figure 6. These results show that the framework’s benefits generalise across materially different regimes rather than reflecting a single favourable environment.

#### 5.4.5. Ablation Study

Table 12 shows what each component contributed; the Transformer reduced the RMSE by 30.3% over the CNN alone, the GP further reduced it by 31.0% while enabling uncertainty quantification (PICP =93.7%), and the entropy-regulated trading module raised the SR by 17.0%, cut the CVaR by 10.3%, and lifted the ESR by 38.9% without affecting prediction accuracy.

#### 5.4.6. Kernel Mixture, BMA, and Transaction Costs

Table 13 shows that the Matérn component dominated for high-volatility stocks (002594.SZ, 000063.SZ) with low $\hat{\nu }$ values, while the SE was stronger for the smoother 600276.SH. The periodic component was uniformly small with $\hat{p}\approx 21$ days (monthly effect). $H_{\mathrm{model}}$ was highest for 600276.SH (1.01 nats, balanced SE and Matérn) and lowest for 002594.SZ (0.82 nats, Matérn-dominant), feeding directly into the EUI for conservative sizing. Table 14 summarises the effect of BMA and transaction cost penalisation on trading performance.

BMA raised the SR by 4.2% and cut the CVaR by 4.2% over the point-kernel baseline, confirming that between-model uncertainty provides an additional risk signal. TC penalisation improved the risk-adjusted metrics at the cost of raw returns (at c=0.3%: net AR −9.2%, SR +7.3%, MDD −8.5%). Full Kelly attained the highest raw return but the worst risk-adjusted performance.

Table 15 further reports the turnover, net returns, and trade counts under different cost rates (four-stock average, full three-year backtest).

#### 5.4.7. Predictive Entropy Dynamics

Figure 6 reveals that (1) the predictive entropy exhibited regime switching between the low-entropy (trending) and high-entropy (volatile) phases; (2) EUI spikes preceded large drawdowns, providing early warning; and (3) the position sizes were inversely correlated with entropy. The Spearman correlation between the daily EUI_*t*_ and next-day absolute return was 0.41 (p<0.001), confirming the entropy measures captured genuine market risk.

## 6. Discussion

The proposed framework integrates three methodological families. Deep learning methods [12,13,15,16] give strong point prediction but lack uncertainty quantification. Here, the CNN and Transformer serve as $f_{\eta }$, gaining uncertainty via the GP. Gaussian process regression [19,20] provides calibrated predictive distributions but struggles with high-dimensional nonlinear features. Here, the GP models only residual autocorrelation in the lower-dimensional *Z*-space.

Using differential entropy, model posterior entropy, and KL divergence as trading signals departs from variance-based measures and offers three advantages: the entropy decomposition (Proposition 1) separates aleatoric and epistemic uncertainty in information-theoretic units, enabling cross-asset comparison; $H_{\mathrm{model}}$ and the inter-model KL capture uncertainty invisible to single-model variance (Table 10); and the link to the Kelly growth rate via mutual information (Remark 9) supplies a theoretical foundation absent in variance-based approaches, extending the entropy-based financial literature [37,38] from descriptive to prescriptive design. Risk management approaches [2,21] require reliable predictive distributions, which Theorems 2 and 3 guarantee.

One concern with a framework spanning deep learning, GPs, information theory, and portfolio optimisation is whether each component has sufficient depth. We address this in three ways. First, the theory was deliberately scoped; identifiability, consistency, and coverage were proven for the specific semiparametric model, giving targeted guarantees that justify entropy-based trading and are unavailable from the components alone. Second, the evaluation added adversarial simulations (Section 5.2.7), sub-period analysis (Section 5.4.4), finite-sample calibration (Table 5), and baseline tuning (Table 9). Third, the integration itself is the main contribution; using the GP predictive entropy as an operational trading signal requires all components together. We acknowledge that deeper investigation of each component, such as non-asymptotic finite-sample bounds, heavy-tailed GP extensions, or multi-output cross-asset modelling, constitutes important future work.

Several limitations should be acknowledged. First, the identifiability result (Theorem 1) relied on independence between *X* and *Z*. When features are shared, partial identifiability may hold, but this requires further investigation. Second, the convergence rate in Theorem 2 depends on the unknown effective dimension $d_{\mathrm{eff}}$; the actual rate may be faster if the true function has additional structure. Third, the CVaR-constrained optimisation assumes Gaussian returns; heavy-tailed extensions using Student *t* processes are a natural direction. Fourth, the current implementation uses independent GP predictions for each asset; a multi-output GP with cross-asset correlations [39] would improve portfolio-level predictions. Fifth, the entropic uncertainty index (EUI) involves tuning parameters γ and δ, which are currently set via cross-validation; an information-theoretic criterion for their automatic selection is an important direction for future work. Sixth, the simulation experiments in Section 5.2 used a data generating process that mirrored the semiparametric structure of the proposed model. While appropriate for verifying theoretical properties, this risks overstating the practical advantage. Section 5.2.7 partially addressed this by showing C-T-GPR remained competitive under misspecified DGPs, but broader adversarial benchmarks (long-memory processes, jump diffusion dynamics, and microstructure noise) would further strengthen the evidence. Seventh, the three-year backtesting window (2022–2024), though longer than the initial one-year design, still covered limited macroeconomic environments. Extending to a full market cycle (e.g., 2015–2024, spanning the 2015 crash, 2018 trade war, and 2020 pandemic) is an important direction for testing robustness under extreme tail events.

## 7. Conclusions

This paper developed an information-theoretic semiparametric framework for entropy-regulated quantitative trading that integrates deep learning, Gaussian process uncertainty quantification, and mathematically grounded trading strategies. The C-T-GPR model decomposes returns into a CNN-Transformer deterministic component and a GP stochastic component with a dynamic mixture kernel, whose identifiability was established in Theorem 1. Theoretical guarantees include consistency of the penalised MLE (Theorem 2) and asymptotic coverage of GP prediction intervals (Theorem 3), linking predictive entropy to risk management. Building on these results, the information-theoretic analysis comprised the predictive entropy decomposition (Proposition 1), model posterior entropy and inter-model KL divergence for kernel uncertainty, and a composite entropic uncertainty index, which fed into an entropy-penalised Kelly criterion (Proposition 2) and CVaR-constrained portfolio optimisation (Proposition 3) with asymptotic reliability guarantees. Empirical validation shows that C-T-GPR achieved the lowest prediction errors with well-calibrated 94.2% prediction interval coverage. Backtesting on a representative four-stock sample drawn from four distinct Chinese A-share industries yielded annualised returns of 15.9–22.4%, Sharpe ratios of 0.49–0.62, maximum drawdowns below 15%, and daily 95%-CVaR reductions of 28–31% relative to the full Kelly baseline. Future directions include multi-output GPs for cross-asset dependence, Student-*t* processes for heavy-tailed returns, sparse GP approximations [40] for high-frequency data, online learning for non-stationary markets, and Rényi entropy generalisations.

## Figures and Tables

**Table 1:** Comparison with representative existing studies across six key dimensions.

Study	Nonlinear	Temporal	Uncertainty	Entropy-Based	Risk-Aware	Theory
	Features	Dependence	Quantification	Measures	Trading	Guarantees
ARIMA [5]		✓				✓
Random Forest [7,23]	✓					
XGBoost and LightGBM [8,9,24]	✓					
LSTM [10,11]		✓				
CNN-BiLSTM-AM [12]	✓	✓				
Transformer [15,16]	✓	✓				
GPR [19]			✓			✓
BNN [17]	✓		✓			
Entropy-XGBoost [27]	✓			✓	✓	
This paper	✓	✓	✓	✓	✓	✓

**Table 2:** Single-run performance comparison on training and test sets.

Metric	C-T-GPR	LASSO	RF	XGBoost	LSTM	GPR
MAE (train)	0.0048	0.0314	0.0245	0.0193	0.0218	0.0396
MAE (test)	0.0117	0.0360	0.0680	0.0661	0.0326	0.0675
RMSE (test)	0.0144	0.0455	0.0531	0.0543	0.0272	0.0551
MRE (test)	0.0347	0.1344	0.1561	0.1566	0.0803	0.1624

**Table 3:** Monte Carlo simulation (M=100 replications): mean (standard deviation).

Metric	C-T-GPR	LASSO	RF	XGBoost	LSTM	GPR
MAE	0.0116	0.0521	0.0714	0.0683	0.0325	0.0706
	(0.0315)	(0.0463)	(0.0578)	(0.0372)	(0.0735)	(0.0775)
RMSE	0.0140	0.0391	0.0564	0.0542	0.0261	0.0555
	(0.0218)	(0.0542)	(0.0661)	(0.0358)	(0.0628)	(0.0859)
MRE	0.0339	0.1138	0.1669	0.1544	0.0762	0.1648
	(0.0336)	(0.0721)	(0.0575)	(0.0462)	(0.0651)	(0.0572)

**Table 4:** Prediction interval quality (95% nominal level, M=100 replications).

Model	PICP (%)	MPIW	MPE (Nats)	Calibration
C-T-GPR	94.2 (1.8)	0.231 (0.034)	−1.42 (0.08)	Well-calibrated
GPR	91.8 (2.7)	0.312 (0.052)	−1.08 (0.12)	Under-covers
MC-Dropout LSTM	87.4 (3.9)	0.287 (0.068)	−1.15 (0.15)	Under-covers
BNN	89.1 (3.2)	0.298 (0.057)	−1.11 (0.13)	Under-covers

**Table 5:** Finite-sample calibration diagnostics (M=100 replications).

*n*	PICP (%)	PICP/0.95	KS Stat	KS *p*-Value
100	89.1 (3.8)	0.938	0.091	0.12
200	91.4 (2.9)	0.962	0.064	0.31
300	93.0 (2.3)	0.979	0.048	0.52
500	94.2 (1.8)	0.992	0.033	0.74
729	94.6 (1.5)	0.996	0.027	0.85

**Table 6:** Performance under different scenarios (Monte Carlo M=100): MAE mean (std).

Case	C-T-GPR	LASSO	RF	XGBoost	LSTM	GPR
T=5	0.021 (0.026)	0.048 (0.041)	0.069 (0.053)	0.065 (0.035)	0.030 (0.069)	0.067 (0.072)
T=10	0.012 (0.022)	0.038 (0.052)	0.055 (0.064)	0.053 (0.035)	0.025 (0.061)	0.055 (0.083)
T=15	0.009 (0.017)	0.030 (0.048)	0.040 (0.059)	0.039 (0.030)	0.018 (0.036)	0.049 (0.044)
T=20	0.013 (0.024)	0.040 (0.053)	0.058 (0.066)	0.055 (0.037)	0.027 (0.063)	0.057 (0.085)
$g_{1}\left(Z\right)$	0.011 (0.020)	0.036 (0.051)	0.053 (0.063)	0.051 (0.033)	0.024 (0.059)	0.053 (0.082)
$g_{2}\left(Z\right)$	0.010 (0.019)	0.043 (0.061)	0.049 (0.051)	0.049 (0.031)	0.023 (0.057)	0.050 (0.079)

**Table 7:** Adversarial simulation: test MAE, mean (std) over M=100 replications.

DGP	C-T-GPR	LASSO	RF	XGBoost	LSTM	GPR
A (Tree)	0.034 (0.028)	0.051 (0.039)	0.022 (0.018)	0.025 (0.021)	0.041 (0.035)	0.058 (0.042)
B (Heavy-tail)	0.027 (0.031)	0.055 (0.048)	0.061 (0.052)	0.058 (0.041)	0.033 (0.038)	0.063 (0.055)
C (Regime)	0.025 (0.024)	0.047 (0.044)	0.052 (0.049)	0.048 (0.037)	0.031 (0.033)	0.054 (0.047)

**Table 8:** Hyperparameter settings of the C-T-GPR model.

Component	Hyperparameter	Value
CNN module	Number of conv layers *N*	3
	Kernel sizes $\ell_{k}$	(5,3,3)
	Channels $c_{k}$	(32,64,64)
	Activation	ReLU
	Pooling stride *s*, window *U*	2,2
SE attention	Reduction ratio	8
Transformer	Number of heads *H*	4
	Hidden dimension *d*	64
	FFN inner dimension	128
	Number of encoder layers	2
	Dropout rate	0.1
GP prior	Kernel	SE + Matérn + periodic mixture
	Initial length scales $\ell_{i}$	median heuristic
	Matérn smoothness ν init	1.5
	Periodic init *p*	21 (days)
	Nugget ω	$10^{-6}$
Optimisation	NN optimiser	Adam ($\beta_{1}\;=\;0.9$, $\beta_{2}\;=\;0.999$)
	NN learning rate	$10^{-3}$
	GP optimiser	L-BFGS-B (max 100 iters)
	$\ell_{2}$ penalty λ	$10^{-4}$
	Batch size	32
	Max outer iterations	200
	Tolerance $\varepsilon_{\mathrm{tol}}$	$10^{-5}$
Trading	Risk aversion κ	0.5
	EUI weights (γ,δ)	(0.3,0.2)
	CVaR level α	0.05
	CVaR bound $\bar{c}$	0.02
	Transaction cost *c*	0.1% (default)

**Table 9:** Baseline hyperparameter search spaces and selected values.

Model	Hyperparameter	Search Space	Selected
LASSO	α (regularisation)	$\left\{10^{-4},10^{-3},\ldots ,10^{1}\right\}$	$10^{-2}$
Random forest	no. of trees	{100,300,500,1000}	500
	max depth	{5,10,15,None}	10
	min samples leaf	{1,5,10}	5
XGBoost	no. of rounds	{100,300,500,1000}	500
	max depth	{3,5,7,9}	5
	learning rate	{0.01,0.05,0.1}	0.05
	subsample	{0.7,0.8,1.0}	0.8
LSTM	hidden units	{32,64,128}	64
	no. of layers	{1,2,3}	2
	dropout	{0.0,0.1,0.2}	0.1
	learning rate	$\left\{10^{-4},5\times 10^{-4},10^{-3}\right\}$	$5\times 10^{-4}$
	epochs	{100,200,300}	200
GPR (standard)	kernel	$\left\{\mathrm{SE},{\mathrm{Mat}é\mathrm{rn}}_{3/2},{\mathrm{Mat}é\mathrm{rn}}_{5/2}\right\}$	Matérn_5/2_
	length scale	median heuristic + MLE	asset-specific
MC-Dropout LSTM	as LSTM above	as LSTM above	as LSTM above
	dropout at test	{0.1,0.2,0.3}	0.2
	MC samples	50	50
BNN	hidden units	{32,64}	64
	no. of layers	{1,2}	2
	prior σ	{0.1,0.5,1.0}	0.5
	variational epochs	500	500

**Table 10:** Trading performance: Proposed entropy-penalised strategy vs. baselines.

Stock	Strategy	AR (%)	SR	Sortino	MDD (%)	Calmar	CVaR_95_ (%)	ESR
000002.SZ	CSI 300	5.18	0.302	0.41	14.62	0.35	2.18	–
	Threshold	15.41	0.478	0.64	14.87	1.04	1.95	–
	Full Kelly	20.63	0.521	0.68	16.21	1.27	2.31	0.38
	Proposed	18.72	0.586	0.82	12.35	1.52	1.59	0.52
000063.SZ	CSI 300	5.18	0.302	0.41	14.62	0.35	2.18	–
	Threshold	12.87	0.398	0.53	15.93	0.81	2.07	–
	Full Kelly	17.82	0.445	0.59	17.45	1.02	2.42	0.31
	Proposed	15.94	0.491	0.71	13.87	1.15	1.71	0.44
002594.SZ	CSI 300	5.18	0.302	0.41	14.62	0.35	2.18	–
	Threshold	18.45	0.517	0.69	13.78	1.34	1.87	–
	Full Kelly	24.91	0.562	0.73	15.63	1.59	2.19	0.42
	Proposed	22.36	0.624	0.89	11.59	1.93	1.53	0.58
600276.SH	CSI 300	5.18	0.302	0.41	14.62	0.35	2.18	–
	Threshold	13.65	0.412	0.55	16.34	0.84	2.12	–
	Full Kelly	18.97	0.458	0.61	17.89	1.06	2.48	0.33
	Proposed	16.81	0.503	0.73	14.12	1.19	1.78	0.46

**Table 11:** Sub-period performance (four-stock average): proposed vs. full Kelly.

Sub-Period	Strategy	SR	MDD (%)	CVaR_95_ (%)
Bear (from January 2022 to October 2022)	Full Kelly	0.18	21.4	3.01
	Proposed	0.31	14.7	2.14
Recovery (from November 2022 to June 2023)	Full Kelly	0.61	11.8	1.87
	Proposed	0.68	9.3	1.42
Range-bound (from July 2023 to December 2024)	Full Kelly	0.42	16.2	2.35
	Proposed	0.53	12.1	1.68
Full sample (from January 2022 to December 2024)	Full Kelly	0.50	16.8	2.35
	Proposed	0.55	13.0	1.65

**Table 12:** Ablation study: contribution of each component (averaged across four stocks).

Configuration	RMSE	PICP (%)	SR	CVaR_95_	Calmar	ESR
(i) CNN only	0.0412	–	0.38	2.21	0.72	–
(ii) CNN + Transformer	0.0287	–	0.43	2.08	0.91	–
(iii) CNN + Trans. + GPR	0.0198	93.7	0.47	1.84	1.12	0.36
(iv) Full (iii + Ent-Kelly + CVaR)	0.0198	93.7	0.55	1.65	1.45	0.50

**Table 13:** Estimated kernel mixture weights and model entropy.

Stock	${\hat{w}}_{\mathrm{SE}}$	${\hat{w}}_{\mathrm{Mat}}$	${\hat{w}}_{\mathrm{Per}}$	$\hat{\nu }$	$\hat{p}$ (Days)	$H_{\mathrm{model}}$
000002.SZ	0.42	0.51	0.07	1.8	21.3	0.92
000063.SZ	0.35	0.58	0.07	1.5	22.7	0.87
002594.SZ	0.31	0.62	0.07	1.3	20.8	0.82
600276.SH	0.45	0.43	0.12	2.1	21.1	1.01

**Table 14:** Impact of BMA and transaction cost penalisation (TC) on trading performance (four-stock average).

Configuration	SR	Sortino	MDD (%)	CVaR_95_	Net AR (%)
Proposed (point kernel, c=0.1%)	0.55	0.79	12.98	1.65	17.21
+ BMA	0.574	0.83	12.41	1.58	17.45
+ BMA + TC (c=0.1%)	0.582	0.85	12.23	1.55	16.89
+ BMA + TC (c=0.3%)	0.591	0.87	11.87	1.51	15.63
Full Kelly (no BMA, no TC)	0.497	0.65	16.80	2.35	20.58

**Table 15:** Transaction cost sensitivity analysis (four-stock average, full sample).

*c* (%)	Turnover	No. of Trades	Gross AR (%)	TC Drag (%)	Net AR (%)	SR (Net)
0.00	1.42	487	18.46	0.00	18.46	0.548
0.05	1.38	471	18.38	0.69	17.69	0.561
0.10	1.29	438	18.21	1.29	16.92	0.572
0.20	1.12	382	17.84	2.24	15.60	0.581
0.30	0.97	331	17.42	2.91	14.51	0.591
0.50	0.74	264	16.73	3.70	13.03	0.583

## Data Availability

The data presented in this study are available on request from the corresponding author. Stock price data were obtained from Tushare (https://tushare.pro, accessed on 13 April 2026).
